# Was King Ludwig II of Bavaria misdiagnosed by Gudden and his colleagues?

**DOI:** 10.1007/s00406-020-01161-8

**Published:** 2020-07-21

**Authors:** Reinhard Steinberg, Peter Falkai

**Affiliations:** Klinik für Psychiatrie Und Psychotherapie, Ludwig-Maximilians-Universität, Nussbaumstr. 7, 80336 Munich, Germany

**Keywords:** Bernhard von Gudden, Ludwig II, Diagnosis, Paranoia (madness), Expert opinion

## Abstract

In 1886, Bernhard von Gudden and three other expert psychiatrists diagnosed the Bavarian King Ludwig II with “paranoia (madness),” a diagnosis that the Bavarian government used to justify removing Ludwig from power. Although Ludwig was not evaluated in detail by the psychiatrists, in their opinion, sworn eyewitness accounts and general knowledge about Ludwig’s behavior provided sufficient grounds for the diagnosis. Ludwig was a great admirer of the musician, Richard Wagner, and shared some of his ideas of an idealistic society. At first, he identified with Wagner’s opera heroes, and he became Wagner’s patron sponsor for life. However, he grew increasingly interested in an absolutist state, envisioning himself as a monarch with a role similar to that of Louis XIV. His multiple building projects, for which he incurred much debt, his conviction that he was descended from the Bourbons through baptism, his increasingly abnormal behavior, and his hallucinations together formed the basis for the psychiatrists’ diagnosis. Although not mentioned in the expert opinion, Ludwig’s homophilic behavior—a scandal at the time—was probably also an important reason for his removal from office. A review of the psychiatric knowledge and societal philosophy of the time indicates that the psychiatrists were correct with their diagnosis in their time.

## Introduction

For more than 150 years, people have questioned whether Ludwig II, the King of Bavaria (1845–1886), was actually ill. An alternative explanation for his behavior is that he was a young man who was certainly eccentric but also highly gifted, not only a patron of the arts but also fond of them, and who was driven into extreme, ultimately fatal isolation by his contemporaries’ lack of understanding. Or, even worse, was psychiatry, acting on behalf of the government, responsible for the King’s death? [1–3].

Most of those who study Ludwig think he was mentally ill [4–6]. However, not even experts fully agree on which illness he might have actually had. In 2011, on the occasion of the 125th anniversary of the death of Ludwig II and his psychiatrist Bernhard von Gudden (1824–1886), historians presented sound publications of the extensive material [7–12]. They emphasized somewhat apodictically, however, that Gudden had misdiagnosed Ludwig [7, 8, 10], probably supported by psychiatric advice from others [2, 4]. The question remains: did Bernhard von Gudden, who was well respected in his time [13], and the other renowned experts, Friedrich Wilhelm Hagen, Hubert von Grashey, and Max Hubrich, really misdiagnose the King on the basis of the knowledge of their time?

When attempting to answer this question, one enters the “minefield of retrospective diagnosis” [14]. Imposing today’s knowledge on the possible medical knowledge of that time often results in a false picture, like the drawing of a caricature. A psychiatric report exists that was prepared by leading experts of the time [15] and that, because the subject of the report was such a prominent figure, has always opened up different lines of argument, including vehemently opposing points of view. A focus on the psychiatric knowledge and societal philosophy of the time should allow the question to be answered more clearly.

## The young king

After the death of Maximilian II in 1864, 18-year-old Ludwig became king, even though he was largely unprepared for the role [10]. The monarch was welcomed with high hopes, and he cut a dazzling figure; however, he soon disappointed the expectations [10, 16]. He was not much of a soldier and became increasingly reluctant to fulfil his political and social obligations and also became increasingly unhappy. He realized his potential mainly in the arts, as a patron of the stage, and as a building contractor.

His first governmental action was to have someone find Richard Wagner (1813–1883) and invite him to Munich. Ludwig had become an early “Wagnerian” and had read his Kunstwerk der Zukunft (The Artwork of the Future). He internalized especially “Tannhäuser” and “Lohengrin,” with whom he almost identified, much to the amazement of the people around him. Early on, in Wagner and his view of art and the state Ludwig saw a person of his own art-oriented nature who also had similar thoughts about the concept of an absolutist state [17, 18]. Wagner was a revolutionary, not only in his musical language but also in his sociopolitical thoughts and actions, and he read Marx and Engels, for example. As contradictory as it may seem, this is where the ideas and ideals of both the 18-year-old Ludwig and the 51-year-old Richard Wagner could be found, and they formed the basis for Ludwig’s friendship with Wagner, his only close, deep friendship [17].

Ludwig had in mind an absolutist monarchy like that of Louis XIV, in which the king shines over everything like the sun as a provider of energy and a role model. Thus, in his imaginings, there would have been no need for either a government or a parliament but only for idealistic co-helpers. This anachronistic idea was opposed by the constitutional monarchical state order imposed by the Bavarian Constitution of 1818 [9]. Under Wagner’s mentorship, Ludwig’s idea of a state became the “art religion,” which should promote a change in the state and its people towards the ideal system of government through moral/musical/artistic education (e.g., they planned to construct a representative Semper/Wagner opera house on the high banks of Munich’s Isar river next to the Maximilianeum, the Bavarian parliament) [17, 18]. Proof that he was quite serious about his ideas is provided by the secret society “Coalition,” the ineffectiveness of which ultimately led to Ludwig’s premature weariness of office and his desire to exchange Bavaria for a kingdom with more submissive subjects (Table 1) [9].

**Table 1:** Medical history of Ludwig II

	Age (years)	Medical history of Ludwig II	Page [23]
August 25, 1845		Difficult birth lasting > 10 h	–
		Family history of psychoses: Alexandra (aunt), Otto (brother)	GA 306
1846	(7 months)	Meningitis, long failure to thrive	–
From 1851	6	Anxious, agitated, shy	GA 307
1864		Joint pain, headaches Becomes King of Bavaria. Friendship with Richard Wagner	–
1866	21	Prussian–Austrian war; Ludwig does not visit his troops Wants to abdicate in favor of his brother Otto, but cannot because Otto is ill	–
1969	24	Groundbreaking for Neuschwanstein, building alterations at Linderhof, plans for Herrenwörth (Schloss Herrenchiemsee) Incomplete break with Richard und Cosima Wagner	–
1870–71	25	German–French war; foundation of the German empire (“Imperial Letter”). Ludwig feels overwhelmed	–
From 1870	25	Secret society “Coalition”: should repeal constitution, depose government Wants to exchange Bavaria for a different kingdom	GA 314
1871/72	23	Avoids contact: only private celebrations of Mass, private performances (“Separatvorstellungen”) at the theater, opera (*n* = 269). Holds court outside Munich, court dinners “… as if he was facing the gallows,” uses privacy screens Alcohol abuse, rants for hours, curses Hardly any contact with the cabinet, mainly keeps company with lackeys, “people from the stables.” Day-night reversal (night rides, celebrations)	GA 307–309
1872	26	Death of Solbrig; Gudden becomes Otto’s physician; isolation of Otto	–
From 1872		Increasingly abnormal behavior	
1876	31	First Bayreuth Festival (“The Ring of the Nibelung”); attends incognito	–
3/1878	33	Ludwig orders “guardianship” for Otto, involuntary commitment	–
1880	34	Wittelsbach 700th jubilee: extreme excitement, permanent ambivalence, does not participate	GA 308
From 1881	36	[Obsessions?] On excursions, dresses up as Ludwig XIV; Chinese court ceremony Image cult: Holy Tree, fence, pillar, statue of Marie Antoinette (“romanticized view”)	GA 314 GA 314
From 1881	36	Hallucinations: sounds, voices (talks to himself when alone in a room, laughs); “imaginary” company at table outside in the snow (with Mme Pompadour, Mme de Maintenon) Motor function: unusual dancing/jumping movements, grimacing, frozen in one posture Affect: irritability, arousal, assaults against > 30 servants; exaggerated affection/abrupt aversion; footman Mayr has to wear a face mask, footman Bucher has to wear sealing wax on his forehead	GA 309 GA 311
From 1881	36	Fantasies of violence: King’s mother, Marie: “Pull on her pigtails, pound on her breasts”; King’s father, Maximilian: “Get his skull from the sarcophagus and box his ears.” Feed the Prussian Crown Prince water and bread and torture him; set fire to Munich, arrest ministers, beat them up; thinks for hours about punishments for “wrongdoers,” etc	GA 315
		Behavior: communicates through gestures (scratching at the door); punishments; servants may not look at Ludwig/his sleigh/food to be served (“Defilement by unseemly looks”); Private orders (“Kammerbefehle”), “boring state business”; Hairdresser Hoppe/Valet Hesselschwerdt are given official tasks: Minister: “Pack, riffraff, rabble…the people do not deserve that I show myself to them…If the chambers [of government] are stubborn, dissolve them, put in others and work on the people.”	GA 312–315 GA 316
1882		[Formal disorders:] obsessive thoughts (e.g., long discussion about table cover), ideas of reference Affect: “Main enjoyment in life is construction,” abdication, suicide	
1885/86		Mortgage lending: “Immediate covering of funds, not an advance, that is disrespectful of me…make the Civil List only available to me again!” Orders bank robbery, burglary; “Nanette Wagner affair” (fraudulent loan of 25 million marks); demanded loans up to 80 million: “The people and their representatives should fulfill their allegiance, whereby they could again garner great favor from his Majesty (“Allerhöchste Gunst”)”	GA 316
From 1884	38	Physical decay: dental status; lack of sleep (chloral hydrate, other hypnotics), headaches (painkillers); neglect. Delusion concerning the Bourbon lineage [19, 22]	GA 318
June 13, 1886	41	Death of Ludwig II and Bernhard von Gudden in Lake Starnberg	–

From: Gudden et al. [15]; Förstl [4]; Steinberg and Hippius [5]; pages numbers in expert opinion are according to Wöbking 1986 [23], parts of medical history not mentioned by Gudden or he is not aware of them

Wagner, royal saxon court music director at the time, was a revolutionary from the barricades in Dresden who had been sought by warrant since 1849. His concept of a state thus clearly differed from that of the young Ludwig II, but his influence on his younger friend made the scandal predictable. When Wagner demanded the dismissal of the ministers Pfistermeister and von der Pfordten (“Pfi and Pfo” [17]), it was the straw that broke the very powerful ministers’ back. Wagner had to leave Munich at the end of 1865 and went to Switzerland. Ludwig gave up, felt the limits of his power and increasingly withdrew into the loneliness of his artistic interests [16–18].

Were Ludwig’s points of view already unsound early in his monarchy? Probably not. But they were generally considered to be anachronistic and dangerous. Ludwig enabled the premiere of “Tristan und Isolde” in 1865, financed the “Ring” and the Bayreuth company and supported Wagner as one of the greatest patrons in the history of music. Any attempt to reduce the relationship between Ludwig and Wagner, which was very close only until 1869, to a giver and taker role or even to a homoerotic relationship would considerably reduce Wagner’s ongoing role in Ludwig’s ideas and artistic desires [17, 18].

## The art world of Ludwig II

Ludwig started with his fantastic castles, which are marveled at today, his self-presentations, the “scenery” of his life. His “identification with Lohengrin,” which annoyed and ultimately led to the loss of his bride ‘Sophie in Bavaria’ because he demanded that she play the role of “Elsa,” Lohengrin’s bride, became tangible with the building of Neuschwanstein (1869), together with the “Tannhäuser world.” Schloss Linderhof (1870), the purest French Rococo, represented Ludwig’s second major theme, the world of the Bourbons, which he acted out in lonely nocturnal sleigh rides dressed up as Louis XIV, even with a scepter and crown. The Schloss Herrenchiemsee (1873) houses a representation of Ludwig’s godfather genealogy, which became increasingly important to him but which he tried to hide from the public [19]. He felt that through his baptism, he was descended from the Bourbons (godfathers in reverse order: King Ludwig II; godfather: grandfather Ludwig I; godfather of the Bourbon Louis XVI; back to Louis IX, the “saint”). Herrenchiemsee is an unfinished but faithful copy of the Palace of Versailles, although it is ~10% larger in almost all dimensions. In contrast to the expected purpose, the furnishings do not serve the “Gloire” (“glory”) of the Wittelsbachers but rather that of the Bourbons from Louis XIV to XVI, Marie-Antoinette, Madame Pompadour, etc.

Was this art world already outside the norm? Did it border on the paranoid? It probably did! For example, Wagner said to his wife Cosima (November 5, 1869, [20]), “…we will have to give away [our son] Siegfried when he becomes a man, he must spend time with people because he must become acquainted with adversity, romp around and misbehave, otherwise he will become a fantasist, maybe a cretin, like we are seeing happen with the King of Bavaria…” (author’s translation). In the given context, this is indeed a distancing from but not a demeaning of Ludwig; rather, it is a father’s fear expressed in the medical concepts of the time (see also August 24, 1871).

Cosima’s diary [20] often refers to Ludwig. Her rather brief, sober eye witness reports—in contrast to the “effusive, elevated diction” [17] (p 231) of the very extensive and historico-culturally relevant exchange of letters between Ludwig, Richard, and Cosima—portrays the ups and downs of their relationship: on the one hand, Wagner’s material dependence on the king, on the other, the over-idealizing importance of Wagnerian arts and ideas to the king. For Ludwig, an end to his friendship with Wagner, despite many a bitter disappointment, would have amounted to a loss of his “center of gravity” [17, p 232]. However, Cosima’s diary supports the general, early suspicions that Ludwig was ill: it records fears of a swift end to Ludwig’s kingship by early death or madness (September 1, 1871; November 8, 1872; June 14, 1873). His identification with the Bourbon Ludwig XIV is noted with astonishment (November 7, 1872; September 1, 1878; November 27, 1881). In contrast, Cosima appears rather to be shocked when she records reports from the king’s environment, e.g., “Recently, he ordered a dinner for twelve people near Partenkirchen, came alone, greeted the empty seats and sat down” (August 21, 1873; author’s translation). This is similar to the imaginary company at the table in the driving snow in 1881, which was included in Gudden’s expert opinion (see Table 1). She continues: “He also never exited his castles through the doors, but through the windows. What lies in store for us here and how soon?” (author’s translation). The early thoughts of some responsible parties about having to set up a guardianship for Ludwig, but who in the end never ventured to take such a step, are documented (March 19, 1878), as are Ludwig’s lack of interest in women (November 21, 1870) and his homophilic tendencies, which were already being publically criticized (October 17, 1875).

We do not know when the perhaps legitimate but “overvalued” idea of the baptismal lineage from the Bourbons transformed into a systematic delusion. In any case, Ludwig did not attend the 700-year Wittelsbach jubilee in 1880 because of his high state of arousal and highly pathological ambivalence that lasted for months [16]. Witness accounts that give a clear description of him are very rare. The 1882 “interview” by the American journalist and author Vanderpoole, in which Ludwig seemed by and large normal, was very likely a fabrication, like two of his other works [21].

With a rather mysterious aura, Ludwig quoted more and more often a verse from Schiller: “I wish to remain an eternal enigma to myself and others,…” This shortened version rather appears to hide the actual meaning. The literary connoisseur Ludwig quotes Beatrice from Schiller’s “The Bride of Messina*”* (forbidden love): “I do not know them/and I do not want to know them, who call themselves the benefactors of my days…an eternal enigma…” This quote describes Beatrice’s denial of her ancestry [19].

On February 5, 1884, Ludwig was treated by Dr. Franz Carl Gerster, a young doctor and dentist who, at the request of the Bavarian administrative government, was also trained in psychiatry by Charcot in Paris. Because it was no longer possible to use gut strings to attach any kind of artificial teeth to Ludwig’s few remaining molars, Gerster suggested fitting a palate plate. Ludwig immediately asked if Louis XIV also wore a palate plate. Gerster describes with psychopathological competence the 4-h-long night meeting with Ludwig. He determined that Ludwig had an accelerated flow of ideas, flight of thoughts, alogical stringing together of thoughts, delusions, and illusory and hallucinatory phenomena. He informed the responsible authorities, who told him clearly that his strong suspicion of mental illness “was interpreted and branded by all as high treason.” Gerster published his experience anonymously in 1886, using only his first and middle name [8, 22]. Gudden was probably not aware of Gerster’s observations.

## The removal of Ludwig II from office

Plans to remove Ludwig from office began no later than fall 1885. The loans to build his palaces, the uncontrollability of further debts, the behavioral problems, the discontinuation of contact with his government, the holding of court in a manner not befitting his status, and in particular the rumors about a somewhat homophile life with the “Chevaux-legers,” a light cavalry unit, and the transfer of Bavarian cavalry soldiers to the valet service, forced the government to act [8, 9]. The state authorities tried to persuade Ludwig to change his financial behavior or possibly to renounce the throne, but their attempts failed. Nobody was really able to get close to him anymore [7–10, 23].

**Fig. 1:**
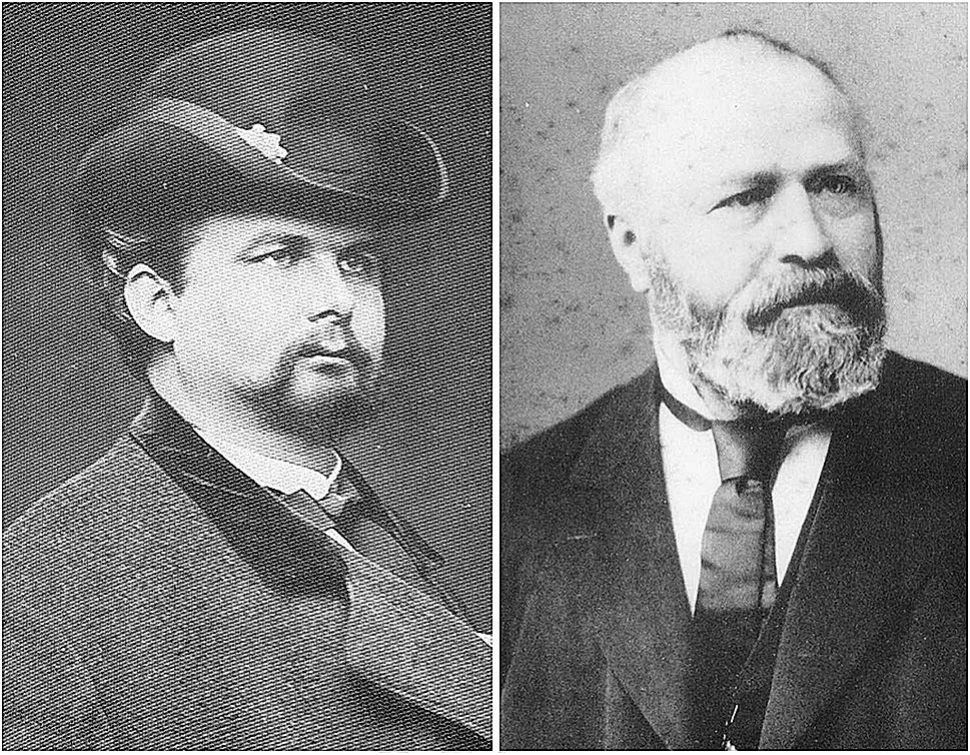
:Left: King Ludwig II. Photograph by Joseph Albert, Munich 1885 (Geheimes Hausarchiv, Wittelsbach Picture Collection, King Ludwig II, 38/46c; Courtesy of Secret Royal Family Archives (Department of Bavarian Central State Archives). Right: Bernhard von Gudden: detail: photograph by Joseph Albert, Munich, 1886 (Stadtarchiv München, DE-1992-C1886076; Courtesy of the City Archives Munich)

Bernhard von Gudden, Professor of Psychiatry at the Ludwig Maximilians University in Munich since 1874 and director of the asylum, was the treating psychiatrist for Ludwig’s brother Prince Otto, who had had schizophrenia since the age of 20 [5, 24]. Therefore, Gudden was entrusted with the royal family’s medical concerns. From March 1886 onwards, on behalf of Prime Minister Lutz, he compiled witness materials for a psychiatric assessment of Ludwig [8] (see Fig. 1). The expert opinion itself was put into writing on June 7, 1886, by Gudden and the other experts at the behest of Prince Regent Luitpold [15]. None of those involved could imagine performing a direct psychiatric examination of the king. The expert opinion reached a clearly stated diagnosis of “paranoia (madness),” which in today’s parlance was most likely schizophrenia. Ludwig was interned 2 days later.

The report lists Ludwig’s abnormalities from birth onwards (Table 1; for the German psychopathological terms see [25] (p 65) (Table 1). Although, almost all the symptoms would be considered today to be clear psychopathological signs of a “paranoia,” various authors have considered most of them to be a misinterpretation of Ludwig’s “normal” behavior because of the extremely great freedom he enjoyed in his role as monarch [1–3]. Hardly any doubts can be raised, however, about the medical testimonies of Dr. Franz Müller, the assistant physician who accompanied Gudden, and the witness statements of Ludwig’s servants affirmed under oath [5, 8, 25], although such doubts have been expressed in the psychiatric literature [2, 3, 26].

The report mentions birth, early childhood development, heredity, then the first early symptoms in Ludwig’s development that may have been psychopathological. No later than 1881, however, behaviors are mentioned that suggest the existence of a psychotic illness not only in the psychiatry of the time. The episode with Gerster in early 1884 suggests that Ludwig’s self-imposed isolation at least played a role in preventing an earlier diagnosis. The symptoms documented from March 1886 on after witness questioning then justify the diagnosis of “paranoia (madness)” [15].

Ludwig’s unstoppable construction-related debt, however, played only a minor role, or it was just a trigger. From a psychiatric perspective, his swiftly changing ideas, ranging from ways to procure loans to giving orders to rob a bank, were part of the paranoid illness. The financial scandal—Ludwig was threatened by an “auction,” i.e., insolvency proceedings before civil courts—was surpassed by Ludwig’s homophilic practices, which were only hinted at between the lines and never explicated, even after his death. The transfer of cavalry soldiers to the valet service was increasingly scandalized in the press [8, 12]. At that time, homophilia was a psychiatric diagnosis, and Section 175 of the German Penal Code regarding homophilic practices between men was valid from 1872 to 1994. The expert opinion omitted these allegations to protect the king. Even after Ludwig’s and Gudden’s death on Pentecost Sunday June 13, 1886, the investigations in the Landtag (state parliament) “went out of their way” to avoid discussing these behaviors, which were considered at that time to be abnormal and indecent and thus punishable [9, 23]. The diagnosis of a “paranoia (madness)” was, therefore, based on the behavioral problems, hallucinations, and paranoid identification with the Bourbons. Only later did Hagen introduce “moral insanity” into the discussion of the differential diagnosis [23]( p 331).

## The psychiatric doctrine of the time

Gudden was considered to be an outstanding academic teacher, scientist, and clinician [27, 28]. He was called upon by the great people of his time, so that they could “…learn psychiatry from him, not just brain anatomy…” [13, 29, 35]. As a co-founder of the retrograde degeneration method (a method also used by Bartolomeo Panizza and Augustus V. Waller, although the three researchers did not know each other), Gudden was an outstanding pioneer of brain research [27, 30], and his students initiated significant developments in psychiatry [13]. Gudden rarely wrote publications and almost all of them were neuroanatomical studies; he did not write a textbook. However, Gudden’s doctrinal system becomes clear in the textbook by Emil Kraepelin (1856–1926), his student and assistant physician from 1878 to 1884. Kraepelin, the founder of today’s psychiatric nosology, published his textbook on psychiatry in 1883—only the first edition of which was entitled “Compendium” [31]—and dedicated it to his teacher “Bernhard von Gudden, in unwavering gratitude.” Each of the subsequent nine editions (1886–1927) contained this dedication. The description of “Primary Madness” in Chapter V of the Compendium (Table 2) corresponds with the structure of the expert opinion (Table 1; [15, 25]). As in chapter VII B (Table 3), “Moral Insanity,” not only the change but also the consistency is impressive compared with today’s nosology.

**Table 2:** Kraepelin E (1883) Compendium: Chapter V: primary madness [31], p 284–312

Kraepelin, Compendium of Psychiatry [31]		Gudden et al. [15]
V. Primary madness		+
(A) Definition	Acute Chronic	− +
(B) Heredity	Congenital, predisposing (original madness) Acquired (unitary psychosis)	+ −
(C) 1. Delusions	Illusions/ hallucinations and illusions of apperception	+
2. Primordial delusion	Sudden delusional ideas, unchanging, delusional system (nothing is a coincidence)	(+)
(a) Depressive delusion	Sinfulness, guilt, abnormal indecision, suicidality	+
(b) Persecutory delusion	Suspicion, shyness, irritability, withdrawal	+
(c) Delusion of grandeur	Focus is on own personality	+
(d) Delusion of ancestry	Not the child of one’s parents	(+)
(e) Religious delusion	Feeling of rapture, transfiguration, enlightenment	+
(f) Delusion of love	Infatuated character, often platonic, secretiveness, masturbation	(+)
(D) Alcoholism	Later predisposing moment	(?)
(E) Degeneration	Skull shape, puerile appearance, juvenile spasms, irritability	+
(F) Course	Acute: good course Chronic: “Prognosis…extremely poor”	− +
(G) Treatment	Shielding from stimuli, mental hospitals, family care, electrotherapy	+

Level of agreement with the expert opinion by Gudden et al. [15]

+,  Extensive; (+),  probable;−,  absent

**Table 3:** Kraepelin E (1883) Compendium: chapter VII B: moral insanity [31], p 351–354

Kraepelin, Compendium of psychiatry [31]		Gudden et al. [15]
VII. Mental disabilities (B) Moral insanity (moral delusion, moral insanity)		
(a) Definition	Disorder of inner life and actions although intelligence is “normal” “To the untrained eye, often a morally bad individual” Thoughtless satisfaction of his egoistic tendencies	(−)
(b) Heredity	Congenital, but also after e.g., head injury, alcoholism Deep, organic-degenerative explanation	(?) (?)
(c) Symptoms	Lack of compassion, abuse of animals as an adolescent, Early sex drive, early masturbation, sexual excesses Wildness, covertness, deceitfulness, craftiness Acts of violence, lies and deception, theft, riots Lack of a sense of honor and attachment to parents/siblings Pronounced selfishness	− ? − ? ? (−)
(d) Intelligence	“Normal,” certain cunning, disorder is not very evident “Blurry” abstraction possible Like in Italian psychiatry: “Born criminal” [Lombroso; 19]	+ − −
(e) Course	Stable condition, but depends on demands on “moral ability”	−
(f) Prognosis	Substantial change not to be expected	−
(g) Treatment	If too sick, then “permanent custody in mental hospital necessary”	?

Level of agreement with the expert opinion by Gudden et al. [15]

+, Extensive; (+), probable; ?, questionable;−, absent

The term “démence précoce” (Morel 1860) was not yet in use in German psychiatry; paranoia only became “dementia praecox” under Kraepelin in 1893. Bleuler then coined the term “schizophrenia” in 1908. In the “degeneration doctrine,” Gudden’s contemporaries discussed possible connections between psychological performance requirements and the price that would have to be paid for them [32, 33]. Kraepelin dropped this complex construct, without comment, in the 7th edition of his textbook (1904). Thus, in the end, Lombroso’s hypotheses did not prevail [34]. The degeneration doctrine defines “moral insanity,” the traces of which can be found today in the psychopathy doctrines.

If one considers Ludwig’s symptoms, which developed with a clear crescendo, one can find traces of sociopathy in his increasing avoidance of contact. The fact that he showed empathy not only at a young age is apparent from his relationship with his schizophrenic brother Otto [5, 24], and even more so from his dealings with Richard and Cosima Wagner. The definition in the Compendium provides no convincing confirmation that Ludwig had “moral insanity.” One could consider a “dissocial psychopathy,” but only as it is defined today [4]. The existing material does not allow one to attribute Ludwig’s character traits primarily to “moral insanity.”

The frequent allegation that Ludwig was not properly evaluated is not completely true. Gudden performed an unplanned assessment when the commission he was in charge of took Ludwig into custody in Neuschwanstein on the night of June 12, 1886. Because of Ludwig’s acute suicidal behavior—he threatened to jump from the tower—the first contact with him, originally planned for 4 a.m., had been brought forward to midnight. Between then and the scheduled departure with the police at 4 a.m., Ludwig and Gudden spoke for almost 4 h [23]. Gudden found confirmation for all the psychopathological symptoms he had written in the expert opinion [28]. Whether he wrote down the findings, which he described to Grashey, in the remaining almost two days of his life, is unknown.

In a statement in Der Nervenarzt in 2019, Haefner [26], one of the representatives of the opinion that Ludwig II did not have psychosis, contradicted Steinberg’s reasoning [25]. After his remarkably extensive studies in 2008 [2], Häfner decided on the diagnosis of a “building frenzy” in sociophobia, even hinting at Gudden’s willingness in his expert opinions to comply with public interests. In 2011 [3] and 2019 [26], Häfner repeated his views, including his view that a king’s servants could not provide reliable evidence about his master. This view must be firmly rejected [28]. According to general legal opinion, witnesses are assessed on the basis of their credibility, not their social position. All of the witnesses from Ludwig’s environment who were included in the expert opinion were sworn in, i.e. clear criteria were used to check their credibility [23] (p 61f).

The few days of direct contact with Ludwig reaffirmed Gudden’s diagnosis [15], and a few hours before his and Ludwig’s death, he telegraphed his views to the state authorities in Munich. Contrary to Häfner’s opinion [26], the telegram should not be considered a “lie” but rather a misjudgement that ended tragically [28]. When changing his role from expert reviewer to treating physician, Gudden underestimated Ludwig’s suicidal tendencies, a mistake that cost him and Ludwig II their lives [13, 28].

## Conclusions

Today, extensive, partly new information is available. Ludwig’s autopsy revealed that the purulent meningitis he suffered as a 7-month-old infant, which was followed by a long failure to thrive, left behind scars in the frontal lobes, and that he also showed frontotemporal atrophy, which could be the basis for an incipient organic delusional psychosis [4, 23] (p 325f). Of course, Gudden was not aware of the autopsy findings. Even with this knowledge, the other experts did not change their opinion and confirmed the paranoia.

Ludwig’s primary disease could, therefore, tentatively be perceived as “frontotemporal dementia” (Pick’s disease, 1892), possibly also as “schizotypal personality disorder” [4]. The latter is very close to schizophrenia. The “Caesarean delusion” [6] that was also discussed was not operationalized, because there were too few occurrences; most notably, Ludwig did not seek out public life, which would have had to be the case to achieve any level of morbid gain. From a modern perspective, wanting to reduce the pathology of Ludwig II to a “building mania” with social phobia features [2, 3, 26] would appear to misread the situation from a psychiatric standpoint. In their day and with their knowledge, Bernhard von Gudden and the other experts were right with their diagnosis of “paranoia (madness),” and they did not misdiagnose Ludwig. There is no justification for alleging that Gudden was a driving force in the process to remove Ludwig II from office or even that Gudden had a treasonous attitude [1–3, 26]. Furthermore, the Bavarian royal tragedy would become neither more understandable nor more bearable if we assumed that the four experts misdiagnosed Ludwig.
